# Diagnostic Accuracy of Procalcitonin in the Diagnosis of Sepsis in Cancer Patients Hospitalized for Infection

**DOI:** 10.1002/cnr2.70384

**Published:** 2025-11-05

**Authors:** Veronica Salvatore, Antonella Viola, Alessandra Spezzano, Alessandra Aquilino, Lorenzo Barili, Mariapia Caprino, Maria Floresta, Giulia Momoli, Alessandra Romiti, Giulia Scurria, Martina Sirna, Alexandro Paccapelo, Margherita Nannini, Andrea Ardizzoni, Fabrizio Giostra

**Affiliations:** ^1^ Emergency Department IRCCS Azienda Ospedaliero‐Universitaria di Bologna Bologna Italy; ^2^ Research and Innovation Unit IRCCS Azienda Ospedaliero‐Universitaria di Bologna Bologna Italy; ^3^ Medical Oncology IRCCS Azienda Ospedaliero‐Universitaria di Bologna Bologna Italy; ^4^ Department of Medical and Surgical Sciences (DIMEC) University of Bologna Bologna Italy

**Keywords:** cancer, diagnosis, procalcitonin, sepsis, sofa score

## Abstract

**Objectives:**

Sepsis is defined as a life‐threatening, dysfunctional body‐response to infection. Procalcitonin (PCT) is considered a marker of sepsis due to bacterial infections and it has been extensively used as a guide to antimicrobial management in the general population. The clinical role of PCT in cancer patients admitted to the Emergency Department (ED) for infection is still little researched.

**Methods:**

A prospective observational study enrolling all adult patients hospitalized for infection referred to the ED of IRCCS Azienda Ospedaliero‐Universitaria di Bologna between February 1st, 2023 and July 31st, 2023 was conducted. The primary endpoint was to evaluate the accuracy of PCT in the diagnosis of sepsis (defined according to the latest guidelines) in patients with cancer in comparison to non‐cancer patients.

**Results:**

1041 out of 1125 eligible patients were enrolled (559 males and 482 females), out of whom 289 (27.8%) had active cancer. PCT levels differed between cancer and non‐cancer patients (1 ng/mL with IQR 5.85 vs. 0.6 ng/mL with IQR 2.7; *p* < 0.001). The AUROC of PCT for the diagnosis of sepsis in the entire enrolled population was 0.717 (95% CI 0.683–0.745), whereas it was 0.655 (95% CI 0.592–0.718) in cancer patients and 0.743 (95% CI 0.708–0.778) in non‐cancer patients (*p* = 0.016). A PCT cut‐off of 0.5 ng/mL (PCT ≥ 0.5 ng/mL) confirmed its accuracy for predicting sepsis in non‐cancer patients (sensitivity 71.5%, specificity 64.1%) but the specificity fell to 44.7% in cancer patients, although sensitivity remained good (sensitivity 78.9%). Conversely, a higher PCT cut‐off of 1 ng/mL, as the most accurate threshold identified in the present study in the cancer population, showed a sensitivity of 66.9% and specificity of 61.2% in predicting sepsis in cancer patients.

**Conclusion:**

Our study confirms the clinical role of PCT as a part of the diagnostic algorithm for sepsis but its diagnostic role is sub optimal in cancer patients.

## 1 Introduction

Sepsis is defined as a life‐threatening, dysfunctional systemic response to infection [1]. The diagnosis of sepsis is confirmed by the acute change of two points in the Sequential Organ Failure Assessment (SOFA) score which was developed to describe quantitatively and objectively the degree of organ dysfunction/failure over time [2, 3, 4]. The incidence of sepsis is continuously increasing [5, 6]. This has led the World Health Organization (WHO) to define sepsis as a global health priority [6]. This demonstrates how sepsis, despite the progressive improvement and cutting‐edge of therapies, determines a high risk of in‐hospital mortality [6, 7, 8, 9]. Procalcitonin (PCT) is a biomarker that has served as an indicator for bloodstream infections and has been used as a guide to antimicrobial management in sepsis and bacterial infections in both the general population [10, 11, 12, 13] and in cancer patients with and without neutropenia [14]. Actually, PCT is widely tested in the Emergency Department (ED), being extremely useful in correctly identifying patients with infection and sepsis; despite an elevation of PCT can occur in conditions other than bacterial infection [15, 16]. Many studies demonstrate that, in the case of blood PCT values below 0.5 ng/mL, the probability of bloodstream infection is drastically reduced, helping clinicians with its good negative likelihood ratio [17, 18]. If the role of PCT as a reliable marker of sepsis has been settled, its clinical utility for sepsis diagnosis in the oncological population needs to be more investigated. Indeed, it has been already shown that in cancer patients PCT levels can be affected by other factors including the type of tumor, and, above all, cancer‐related chronic inflammatory states. In some studies, a negative prognostic value of increased levels of PCT in end‐stage cancer patients has also been shown [19, 20, 21, 22]. A previous paper evaluated the role of PCT in critically ill patients with cancer admitted to the Intensive Care Unit (ICU) whilst our purpose is to evaluate all patients admitted to the hospital for infections other than viral ones [23]. The identification of sepsis in the ED is mandatory in order to decide an eventual hospital discharge or a patient's allocation in the best setting and a quick start of an appropriate antibiotic therapy. Decisions have to be rapidly taken and the suspicion of sepsis is often made even in the absence of SOFA calculation. Thus, the aim of the present study was to evaluate the accuracy of PCT in the diagnosis of sepsis in ED patients with cancer in comparison with non‐cancer patients.

## 2 Materials and Methods

The present prospective observational study includes all adult patients referred to the ED of IRCCS Azienda Ospedaliero‐Universitaria di Bologna who were hospitalized for infection between February 1 2023 and July 31 2023. The diagnosis of infection was made in case of clinical suspicion of disease caused by microorganisms typically characterized by fever, an increase in white blood cells and C‐reactive protein. Patients were continuously assessed to determine if other diagnoses were more likely in order to exclude those initially diagnosed with infection that turned out to have non‐infective diseases. The reference standard for the diagnosis of sepsis was performed according to the SOFA score as suggested by the Surviving Sepsis Campaign [3]. In particular, sepsis was defined as a SOFA score ≥ 2 or 2 points above the baseline SOFA score of patients before the infectious event. Baseline SOFA was extracted from online medical records, including the latest blood tests available in the patient's medical records or obtained from their clinical history. Patients with viral infections (including Sars‐Cov 2 co‐infection) were excluded. Imaging studies, laboratory exams and microbiological findings (at first medical contact in the ED) were collected. The following data were specifically recorded: complete blood count, creatinine, urea, sodium, potassium, C‐reactive protein, PCT, aspartate aminotransferase, alanine aminotransferase, prothrombin time, total and fractioned bilirubin and arterial blood gas analysis. PCT values were quantified in plasma by electro‐chemoilluminescence immunoassays (Roche Diagnostics GmbH, Mannheim, Germany) considering normal values < 0.05 ng/mL, the cut‐off usually used to rule out sepsis [15]. Clinical parameters were merged in the National Early Warning Score (NEWS‐2), which included respiratory rate, body temperature, systolic blood pressure, heart rate, oxygen saturation, level of consciousness, the presence of hypercapnic respiratory failure, and the eventual use of supplemental oxygen [24]. The Charlson Comorbidity Index was calculated for classifying comorbidities [25]. The presence of an active tumor was recorded. In case of a negative 1‐year follow‐up after curative treatment, patients were not categorized as cancer patients. The evidence of metastases was also recorded by evaluating electronic medical records. Written informed consent was provided by patients or legal representatives within 72 h of hospital admission and before study enrollment. The patient's family members or closest relatives cannot provide informed consent to participate in the study according to local guidelines. Deceased patients within 72 h of hospital admission were also included. The study was approved by the local ethical committee (Comitato Etico Area Vasta Emilia Centro: CE‐AVEC n° 637/2022/0ss/AOUBo), according to local guidelines. A study grant has been awarded by the Italian Ministry of Health (Ricerca Corrente 2022–24, codice progetto RC‐2024‐2 790 047), without any role in results analysis and discussion.

### 2.1 Statistical Analysis

Patient demographic and clinical characteristics were reported as frequencies and percentages for categorical variables and as mean and standard deviation (SD) or median and interquartile range (IQR) for continuous variables. Continuous variables were compared using the Student t test or Mann–Whitney U‐test depending on data distribution. Normal distribution was verified with the Kolmogorov–Smirnov test. Qualitative variables were analyzed using 2 × 2 contingency tables and Fisher's exact test. The accuracy of PCT in the diagnosis of sepsis was assessed using Receiver Operating Characteristic (ROC) analysis and calculating related Area Under ROC (AUROC), sensitivity, specificity, positive predicted value (PPV) negative predicted value (NPV), positive likelihood ratio (LR+) and negative likelihood ratio (LR‐). Youden's test was used to identify the best threshold in cancer patients. The asymptotic z test was used for testing H_0_: AUROC = 0.5 and to compare AUROC values. The *p*‐value was considered significant when less than 0.05 for two‐tailed tests. Statistical analysis was performed using IBM SPSS Statistics for Windows software, Version 28.0 (Armonk, NY: IBM Corp).

### 2.2 Sample Size Calculation

A single‐group diagnostic test design was considered for the sample size calculation, aiming to obtain two‐sided 95% confidence intervals for the sensitivity and the specificity. The simple asymptotic formula was used to calculate the confidence interval limits. The sample sensitivity and specificity were assumed to be 0.7, and the prevalence was assumed to be 0.5. To produce sensitivity and specificity confidence intervals with a width of no more than 0.08, at least 1010 subjects were needed.

With 1041 patients, a prevalence of 50.4%, a sensitivity of 73.4%, a specificity of 58.3%, we obtained a 95% confidence interval of 0.076 for sensitivity and 0.085 for specificity.

## 3 Results

A total of 1125 patients were potentially eligible. However, written informed consent was not collected in 84 patients for study participation rejection or patients' clinical conditions that avoided a valid informed consent. A total of 1041 patients with infections were thus enrolled (559 males and 482 females), of whom 289 (27.8%) had active cancer. The diagnosis of sepsis was made in 520 patients (50%) and 170 (16.3%) died during hospitalization. The length of hospitalization was 10 days (11) in the whole population. Respiratory tract infection was present in 34% of patients, urinary tract infection in 27.9% abdominal infection in 11.1%, soft tissue infection in 8.1%, bone or joint infection in 2.1%, central vein catheter‐related infection in 1.4% of patients. In 110 patients (10.6%) the source of infection remained unknown and in 4.9%, it was other than the above reported. Among the cancer patients subgroup, 43 (14.9%) were affected by pulmonary cancer, 84 (29.1%) by hematological malignancy, 25 (8.7%) by pancreatic cancer, 21 (7.3%) by breast cancer, 52 (18%) by urinary tract cancer, 16 (5.5%) by intestinal cancer, 13 (4.5%) by gynecological cancer, 11 (3.8%) by skin/soft tissue cancer, 6 (2.1%) by liver cancer, 8 (2.8%) by brain cancer, 2 (0.7%) by thyroid cancer and 8 (2.8%) by cancer from other origins. Patients' characteristics are reported in Table 1. Considering the whole population included, a statistically significant difference in PCT levels between septic and non‐septic patients was found (1.6 ng/mL vs. 0.3 ng/mL, *p* < 0.001).

**TABLE 1:** Baseline characteristics of non‐cancer and cancer patients.

	Non‐cancer patients (752)	Cancer patients (289)	*p*
Sex (Male/Female)	402/350	157/132	n.s.
Age	79 (34)	74 (19)	< 0.001
Charlson comorbidity index	5 (3)	7 (4)	< 0.001
Systolic arterial pressure (mmHg)	125 (31)	115 (30)	< 0.001
Diastolic arterial pressure (mmHg)	70 (20)	65 (15)	< 0.001
Heart rate (beat/min)	95 (28)	98 (29)	n.s.
Respiratory frequency (breath/min)	17 (6)	17 (4)	n.s.
WBC (10/L)	12.8 (7.9)	10.65 (9.94)	0.01
Neutrophils (10^9^/L)	10.7 (7.7)	8.28 (9.1)	< 0.001
Platelets count (10^9^/L)	227 (130)	205 (153)	< 0.001
Creatinine (mg/dL)	1.20 (1)	1.10 (1)	n.s.
Total bilirubin (mg/dL)	0.8 (0.6)	0.8 (0.7)	n.s.
Lactate (mmol/L)	1.4 (1.6)	1.3 (1.5)	n.s.
Procalcitonin (ng/mL)	0.6 (2.7)	1 (5.85)	< 0.001
C‐reactive protein (mg/dL)	11.6 (13.6)	11.9 (14)	n.s.

*Note:* Data are reported as mean (sd); n.s.: non‐significant; WBC: white blood cells.

**FIGURE 1:**
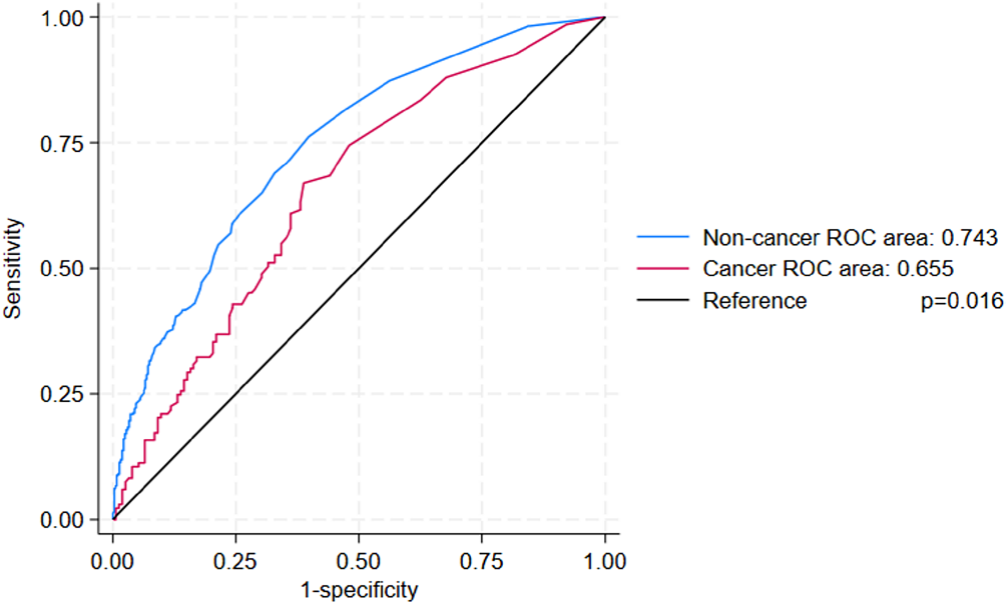
Procalcitonin AUROC plot for cancer (red line) and non‐cancer (blue line) patients.

The AUROC of PCT for the diagnosis of sepsis was 0.717 (95% CI 0.683–0.745). The PCT threshold value of 0.5 ng/mL had a sensitivity of 73.4% and a specificity of 58.3% in the diagnosis of sepsis. When comparing PCT levels between non‐cancer and cancer patients, it differed between cancer and non‐cancer patients (1 ng/mL with IQR 5.85 vs. 0.6 ng/mL with IQR 2.7; *p* < 0.001). The accuracy of PCT for the diagnosis of sepsis in terms of sensitivity, specificity and AUROC within each subgroup is listed in Table 2. In particular, the AUROC of PCT for the diagnosis of sepsis in cancer patients was 0.655 (95% CI 0.592–0.718, *p* < 0.001), while it was 0.743 (95% CI 0.708–0.778, *p* < 0.001) in non‐cancer patients (Figure 1). When comparing the AUROC value in the two populations, we found significant differences (*p* = 0.016). The accuracy of the PCT cut‐off of 0.5 ng/mL (PCT ≥ 0.5 ng/mL) for predicting sepsis in non‐cancer was confirmed, with a sensitivity and specificity of 71.5% and 64.1%, respectively. Conversely, in cancer patients, the specificity of this cut‐off resulted lower, at 44.7%, although sensitivity remained at 78.9%. A higher PCT cut‐off of 1 ng/mL, as the most accurate threshold identified in the present study in the cancer population, showed a sensitivity of 66.9% and specificity of 61.2% in predicting sepsis in cancer patients. On the contrary, the same cut‐off demonstrated low sensitivity (58.8%) despite good specificity (75.8%) in predicting sepsis in non‐cancer patients.

**TABLE 2:** Accuracy of procalcitonin in diagnosing sepsis at different cut‐offs in different subgroups.

	PCT ≥ 0.5 ng/mL						PCT ≥ 1 ng/mL						AUROC
	Sens.	Spec.	PPV	NPV	LR+	LR‐	Sens.	Spec.	PPV	NPV	LR+	LR‐	
All patients	73.4	58.3	64.1	68.3	1.76	0.46	60.9	71.4	68.4	64.3	2.13	0.55	0.717
													(0.683–0.745)
Non‐cancer patients	71.5	64.1	68.1	67.6	1.99	0.44	58.8	75.8	72.3	63.1	2.43	0.54	0.743
													(0.708–0.778)
Cancer patients	78.9	44.7	55.6	70.8	1.43	0.47	66.9	61.2	60.1	67.9	1.72	0.54	0.655
													(0.592–0.718)
Non‐metastatic	76.3	48.8	55.8	71.0	1.49	0.49	61.8	64.0	59.5	66.7	1.72	0.60	0.658
													(0.576–0.741)
Metastatic	83.3	38.7	54.2	72.7	1.36	0.43	74.1	56.0	59.7	71.4	1.68	0.46	0.655
													(0.555–0.755)
Solid cancer	73.9	40.6	53.0	63.2	1.24	0.64	64.6	55.7	56.9	63.4	1.46	0.64	0.610
													(0.532–0.687)
Hematological malignancy	91.0	54.3	61.8	89.3	1.99	0.17	73.0	73.9	69.2	77.3	2.80	0.37	0.761
													(0.657–0.865)

Abbreviations: AUROC, area under receiver operating characteristic; LR+, positive likelihood ratio; LR−, negative likelihood ratio; NPV, negative predictive value; PCT, procalcitonin; PPV, positive predictive value; Spec., specificity; Sens., sensitivity.

**FIGURE 2:**
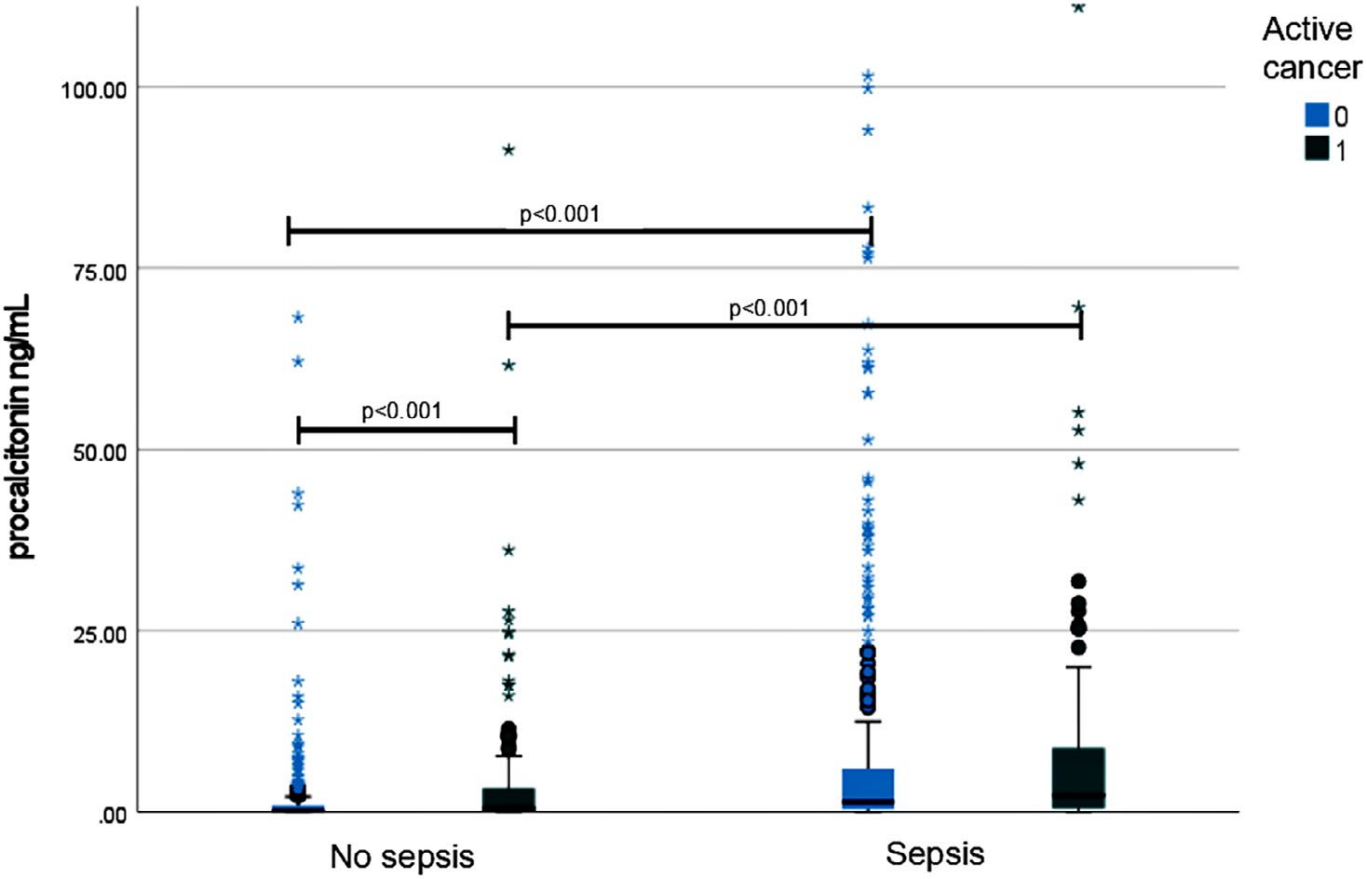
Procalcitonin levels according to the presence of sepsis and cancer. Left box plots represent patients without sepsis and right box plots patients with sepsis, according to the presence of active cancer (light blue) or not (green).

As expected, a significant difference in PCT levels between septic and non‐ septic patients, both in the cancer subgroup (2.3 ng/mL with IQR 8.7 vs. 0.5 ng/mL with IQR 0.8; *p* < 0.001) as well as in the non‐cancer subgroup (1.4 ng/mL with IQR 5.7 vs. 0.2 ng/mL with IQR 0.8; *p* < 0.001) was found (Figure 2). Moreover, among non‐septic patients, a relevant difference in PCT levels between cancer and non‐cancer patients was found (*p* < 0.001). In particular, PCT values were 0.5 0.5 ng/mL (3.2) in cancer patients and 0.2 ng/mL (0.8) in non‐cancer patients respectively.

**FIGURE 3:**
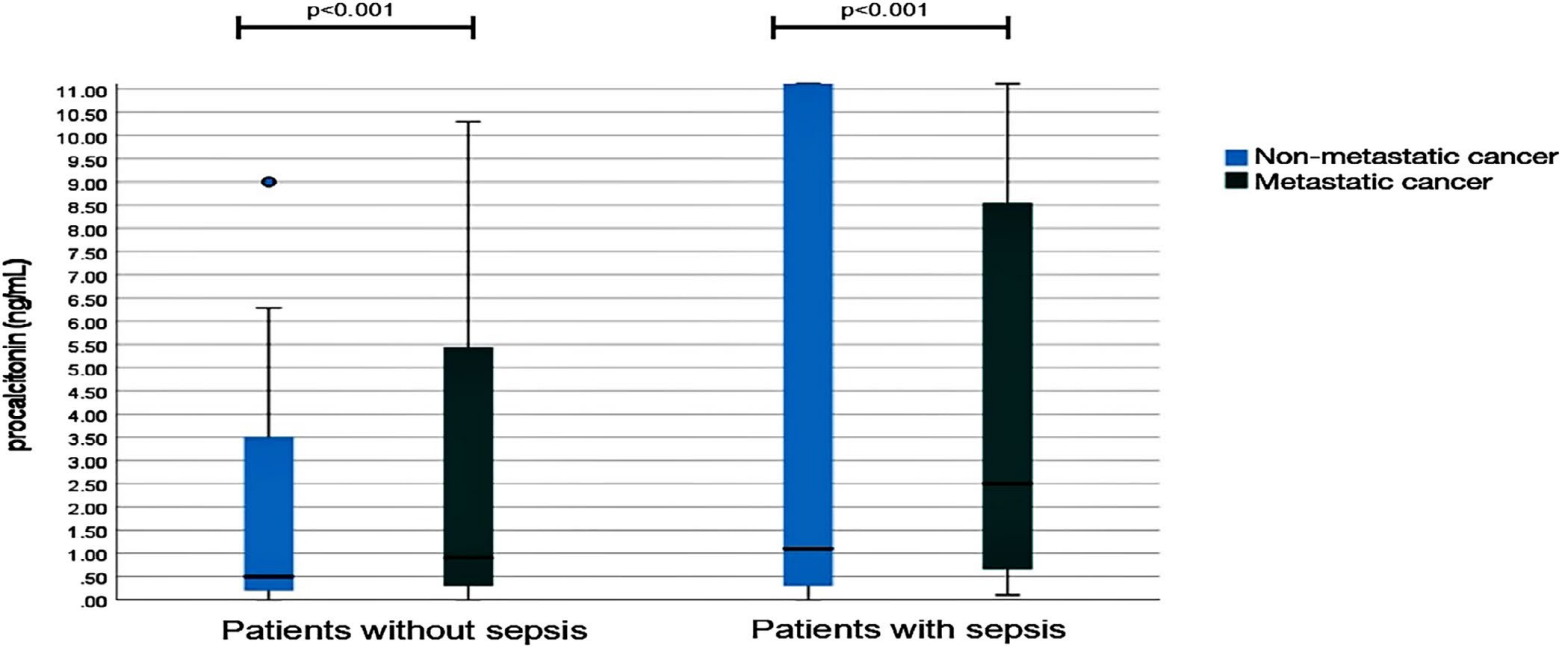
Procalcitonin levels according to the presence of metastases in patients without sepsis and with sepsis. Left box plots represent procalcitonin levels in patients without sepsis and right box plots represent procalcitonin levels in patients with sepsis according to the presence of metastatic cancer (light blue) or not (green) metastatic cancer.

Among cancer patients, the accuracy of PCT for the diagnosis of sepsis was higher for patients with hematological malignancy with respect to patients with solid cancer in terms of AUROC (0.761, 95% CI 0.657–0.865 vs. 0.610, 95% CI 0.532–0.687; *p* = 0.023). Conversely, the metastatic setting did not affect PCT levels, both in non‐septic and septic cancer patients (Figure 3). In particular, among non‐septic cancer patients, PCT levels did not differ between metastatic versus non‐metastatic patients (0.75 ng/mL, IQR 4.9, versus 0.5 ng/mL, IQR 3.1; *p* = 0.07) although values were highly variable. Similarly, the presence of metastases did not affect PCT levels considering only septic patients (*p* = n.s.). The PCT cut‐off of 0.5 ng/mL was even less accurate in predicting sepsis in patients with metastatic cancer (sensitivity 83.3%, specificity 38.7%) with respect to patients without metastatic cancer (sensitivity 76.3%, specificity 48.8%). The PCT cut‐off of 1 ng/mL was confirmed to be more accurate in both subgroups, with a sensitivity and specificity of 74.1% and 56%, respectively in patients with metastatic cancer and of 61.8% and 64% in patients with non‐metastatic cancer.

Lastly, as expected, the presence of sepsis affected mortality (*p* < 0.001).

## 4 Discussion

The present study aims to evaluate the accuracy of PCT in diagnosing sepsis in cancer patients compared to non‐cancer patients. The results show that its diagnostic accuracy is lower in cancer patients with respect to non‐cancer ones, namely due to higher PCT levels even in the absence of sepsis in this population. This concept has been demonstrated in our paper by the comparison between ROC curves of PCT in patients with and without cancer (*p* = 0.016). We also found that the commonly used cut‐off of 0.5 ng/dL for sepsis diagnosis has a low specificity (44.7%) in cancer patients. These results are in line with the recent meta‐analysis by Lee and colleagues where pooled sensitivity of 60% and specificity of 78% have been reported in non‐neutropenic cancer patients [26]. However, in that study the endpoint was the diagnosis of infection, not sepsis, as in our case. Moreover, we demonstrated that a higher cut‐off, namely 1 ng/mL, is more accurate in cancer patients. In everyday clinical practice, the frequent finding of PCT levels higher than 0.5 ng/mL in this population could raise the suspicion of sepsis even in the case of a mild infection. The issue of admitting or not to hospital patients with infection is a main issue in the ED, especially for the widespread use of PCT even in peripheral hospitals or out‐patient clinics. Recognizing the low specificity of PCT for the diagnosis of sepsis in cancer patients may be crucial, in order to spare unnecessary hospitalizations and to avoid unnecessary therapies [16]. Moreover, the finding of high PCT values may lead to the use of “watch” and “reserve” antibiotics, according to the WHO AwaRe classification, even in cases where “access” ones may be appropriated. To the best of our knowledge on this topic, the present study is the only one that has included all patients admitted to the hospital for infection directly from the ED. Some papers have demonstrated that PCT values are higher in cancer patients with respect to non‐cancer subjects, especially in the case of advanced stage of disease. Focusing on infections, only the discrimination between non‐febrile cancer patients and febrile cancer patients and between bacteremia or sepsis using older sepsis definitions has been tested. Cut‐offs higher than 0.5 ng/mL have been suggested to discriminate cancer patients with and without bacterial infection but its role in the discrimination of sepsis with updated criteria has not been already tested [27, 28, 29]. Moreover, a previous paper demonstrated that patients with metastatic tumors had higher PCT values than those without metastases in 112 patients among which the highest PCT value was 1.76 ng/mL in the subgroup of patients with metastatic tumors in the absence of sepsis (9 patients) [30]. Values of PCT in our wide population are more variable, reaching 734 ng/mL in the subgroup of metastatic patients in the absence of sepsis, probably due to the large population included in our study. Concerning patients with hematological malignancies, a value of PCT ≥ 0.54 ng/mL has been demonstrated as accurate in differentiating children with or without bacteraemia alone or in combination with IL10 [31, 32]. Interestingly, our patients with hematological malignancies had PCT values more similar to patients without cancer than to patients with solid cancer, according to the literature [33].

This concept opens the way to further investigating the topic on a wider population. The main limitation of our study is given by the absence of a control group of cancer patients without infection. Furthermore the study enrolled only patients admitted to the hospital. For these reasons, PCT values might be higher than in other populations that include also dismissed patients. However, we considered it unethical to collect blood samples, including PCT, in all patients referred to ED for infection even in the absence of clinical need for them (e.g., cystitis in young women, infected skin cysts, or other mild infections that can be discharged from ED only after medical judgment without laboratory exams). Moreover, the aim of the present study was to investigate the diagnostic accuracy of PCT in patients admitted to hospital from ED for infection, starting from real‐life data.

In conclusion, the present study confirms the role of PCT as a supplementary tool in the diagnosis of sepsis, given the fair accuracy demonstrated in patients without cancer, but urges even more caution in considering cancer patients as septic only on the basis of PCT values. More attention should be paid to clinical assessment and scoring systems such as SOFA score calculation to identify sepsis in cancer patients with infections, in order to allocate them in the appropriate setting and to treat them quickly. Further studies are necessary to explore PCT variability in cancer patients, especially considering the elongation of their life expectancy and resource shortages.

## Author Contributions

V.S. and A.V.: conceptualization; V.S., A.V., A.S. and A.P.: data curation; V.S., A.V., and A.P.: formal analysis; A.V.: funding acquisition; A.S., AlAq, L.B., M.C., M.F., G.M., A.R., G.S. and M.S.: investigation; V.S. and A.P.: methodology; F.G. and AnAr: supervision; V.S., A.V., M.N. and A.S.: writing – original draft; V.S. and A.S.: writing – review and editing.

## Ethics Statement

The study was approved by the local ethical committee (637/2022/0ss/AOUBo).

## Conflicts of Interest

The authors declare no conflicts of interest.

## Data Availability

The data of this study are available from the corresponding author upon reasonable request to the corresponding author. https://doi.org/10.5281/zenodo.14967365.
